# Supplementary material for: A snapshot of the Physcomitrella N-terminome reveals N-terminal methylation of organellar proteins
Source: Plant Cell Rep. 2024 Oct 3;43(10):250. doi: 10.1007/s00299-024-03329-1 (PMC11450134; doi:10.1007/s00299-024-03329-1)
Supplement: Supplementary file 1 — Supplementary file1 (DOCX 1716 KB) [file 299_2024_3329_MOESM1_ESM.docx]

**Supplementary Information**

**A snapshot of the Physcomitrella N-terminome reveals N-terminal methylation of organellar proteins**

Sebastian N.W. Hoernstein^1^, Andreas Schlosser^2^, Kathrin Fiedler^3,*^, Nico van Gessel^1^, Gabor L. Igloi^3^, Daniel Lang^1,4^, Ralf Reski^1,5^

^1^ Plant Biotechnology, Faculty of Biology, University of Freiburg, Schaenzlestr. 1, 79104 Freiburg, Germany

^2^ Rudolf Virchow Center for Experimental Biomedicine, University of Würzburg, Josef-Schneider-Str. 2, 97080 Würzburg, Germany

^3^ Institute of Biology III, University of Freiburg, Schaenzlestr. 1, 79104 Freiburg, Germany

^4^ Bundeswehr Institute of Microbiology, Microbial Genomics and Bioforensics, Neuherbergstr. 11, 80937 Munich, Germany

^5^ Signalling Research Centres BIOSS and CIBSS, Schaenzlestr. 18, 79104 Freiburg, Germany

* Present address: Lonza, Hochbergerstr. 60A, 4057 Basel, Switzerland

**Corresponding author**: ralf.reski@biologie.uni-freiburg.de


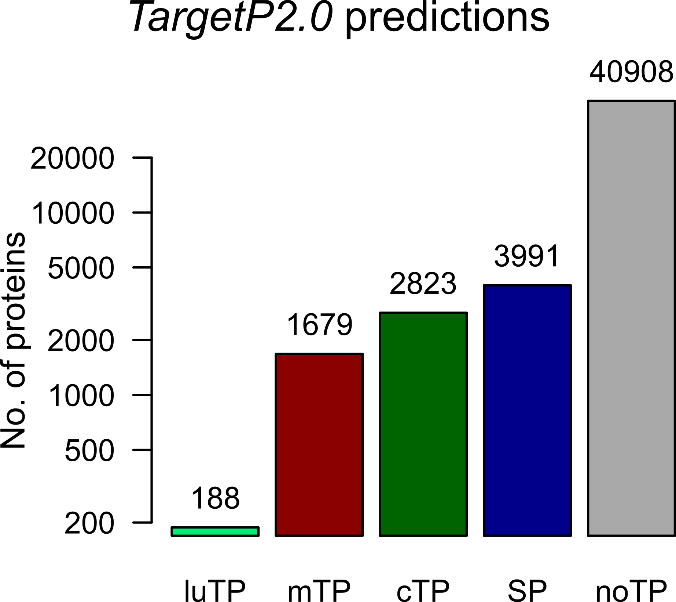


**Figure S1** **Overview on the numbers of cleavable targeting sequences in Physcomitrella predicted by *TargetP2.0*.** Predictions were done on a non-redundant protein isoform list (49,589 entries) of all Physcomitrella V3.3 protein models (Lang et al. 2018). luTP: thylakoid luminal targeting peptide; mTP: mitochondrial targeting peptide; cTP: plastid targeting peptide; SP: (secretory) signal peptide; noTP: no targeting peptide predicted.


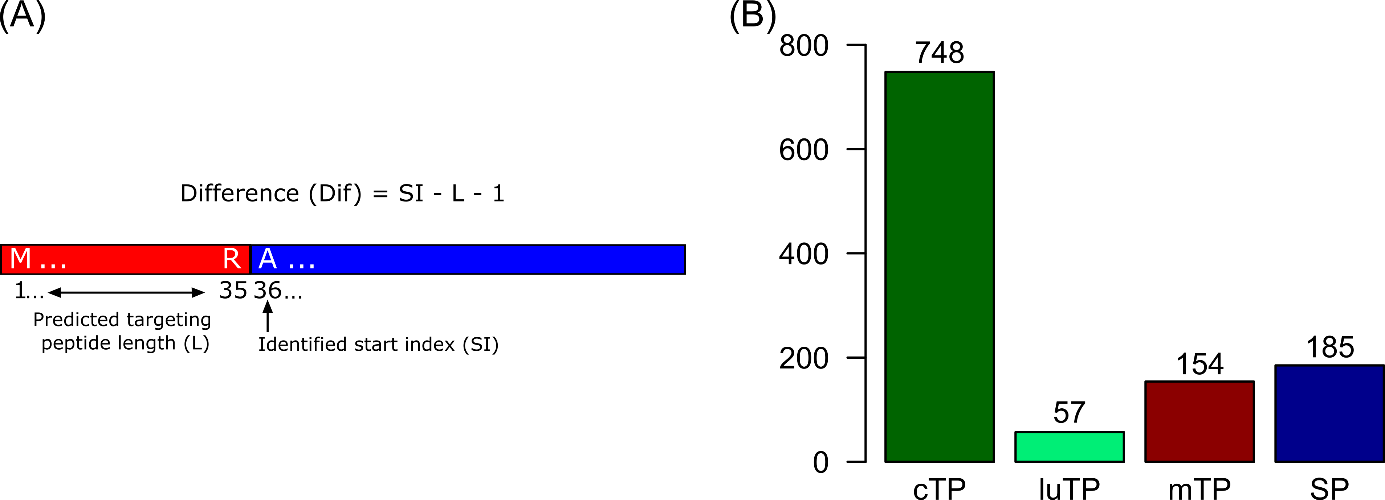


**Figure S2 Overview on targeting peptide cleavage site identification and summary of identified targeting peptide cleavage sites.** (**A**) Scheme depicting the calculation of the difference between predicted targeting peptide cleavage site and experimentally observed N-terminus. A difference of 0 indicates full agreement between prediction and observation. (**B**) Summary of identified plastid (cTP), luminal (luTP), mitochondrial (mTP) and secretory (SP) targeting peptide cleavage sites. A difference of ±5 from a predicted cleavage site was accepted. Data are available from Supplemental Table S4.


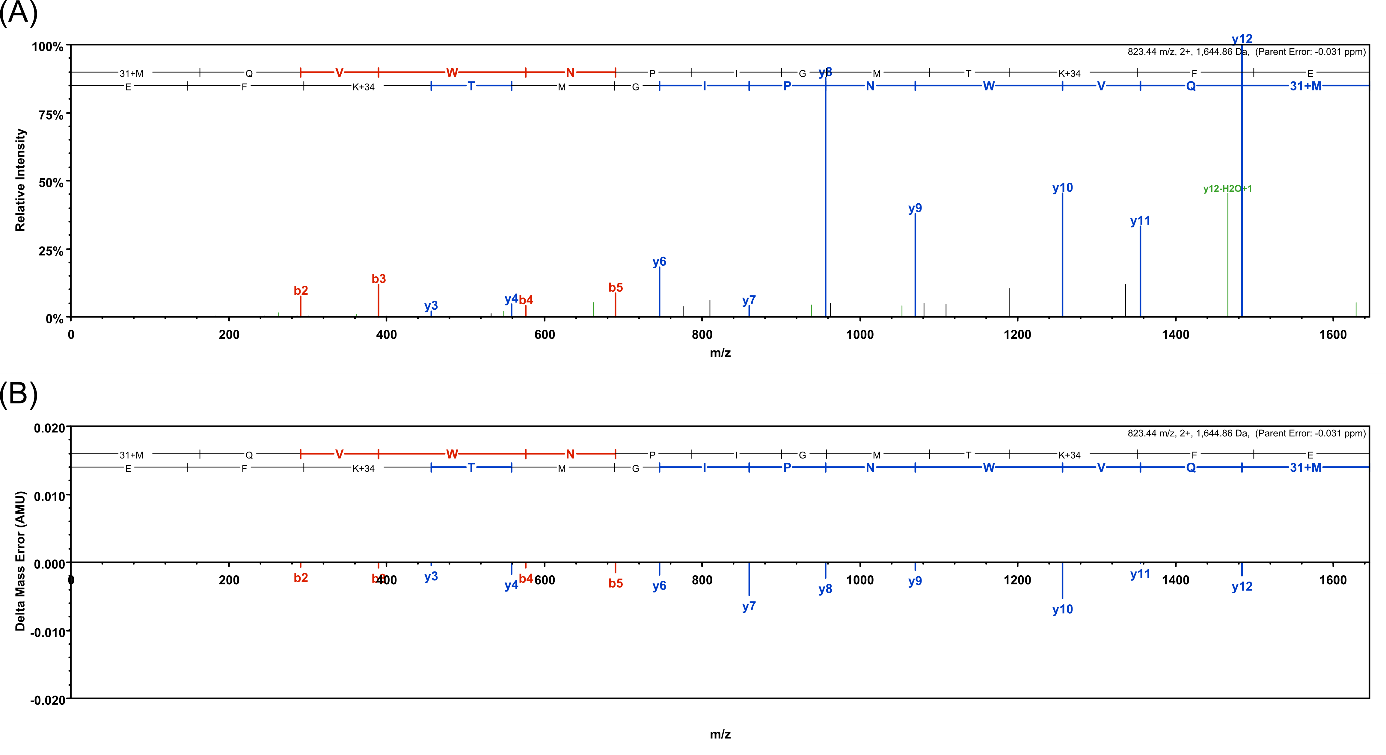


**Figure S3** **Higher-energy collisional dissociation (HCD) fragment mass spectrum and fragment mass error distribution of the identified N-terminal peptide of RbcS (Pp3c12_19890V3.4).** (**A**) HCD fragment mass spectrum of the peptide MQVWNPIGMTKFE. A mass shift of +31 indicates the hybrid modification of a post-translationally incorporated methyl group and the further addition methyl group from the preformed reductive methylation during sample preparation (^13^CD_2_CH_2_, +31.047208). (**B**) Fragment mass error distribution of the b- and y-ion series.


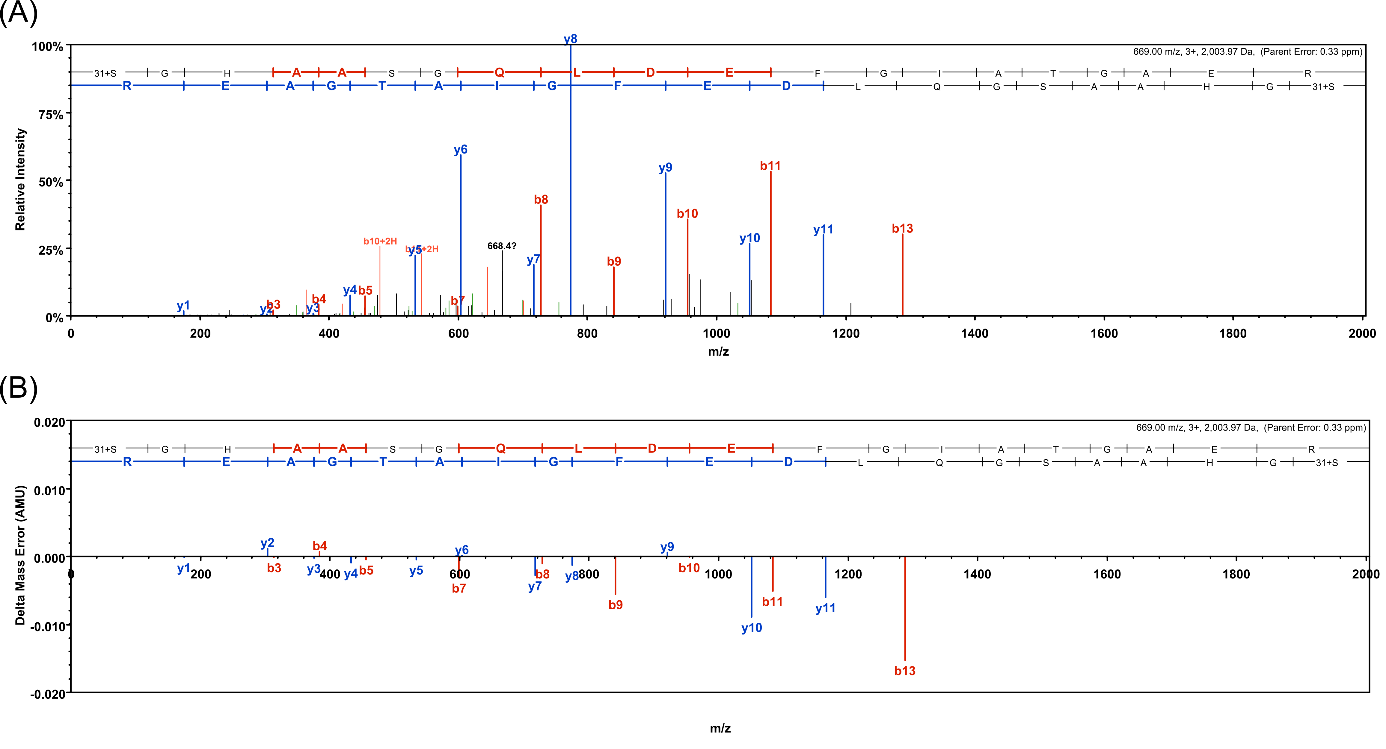


**Figure S4** **Higher-energy collisional dissociation (HCD) fragment mass spectrum and fragment mass error distribution of the identified N-terminal peptide of COX5B (Pp3c19_11870V3.1).** (**A**) HCD fragment mass spectrum of the peptide SGHAASGQLDEFGIATGAER. A mass shift of +31 indicates the hybrid modification of a post-translationally incorporated methyl group and the further addition methyl group from the preformed reductive methylation during sample preparation (^13^CD_2_CH_2_, +31.047208). (**B**) Fragment mass error distribution of the b- and y-ion series.

**
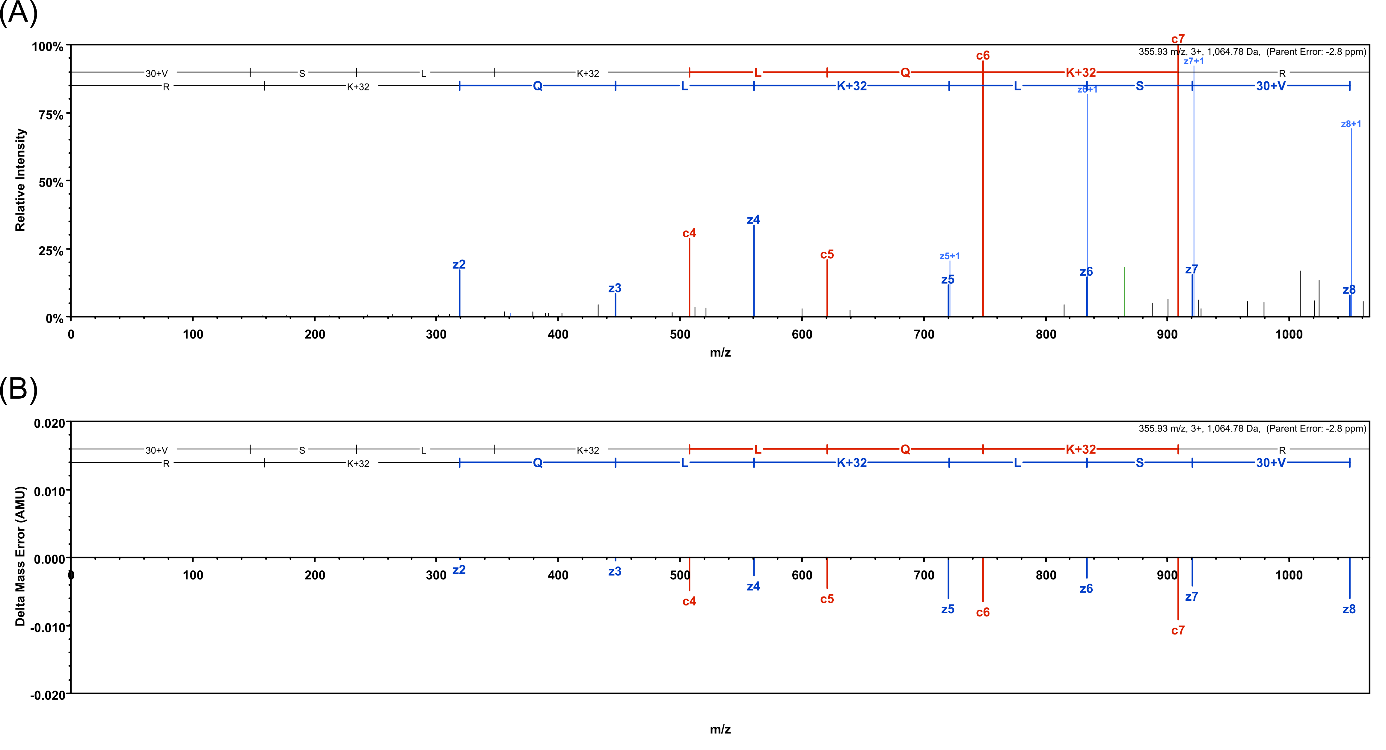
**

**Figure S5** **Electron-transfer dissociation (ETD) fragment mass spectrum and fragment mass error distribution of the identified N-terminal peptide of RPL19 (Pp3c18_14440V3.1).** (**A**) ETD fragment mass spectrum of the peptide GKQISEIKDFLLTAR. A mass shift of +30 indicates the hybrid modification of a post-translationally incorporated methyl group and the further addition methyl group from the preformed reductive methylation during sample preparation (C_2_D_2_H_2,_ +30.043854 Da). (**B**) Fragment mass error distribution of the b- and y-ion series.


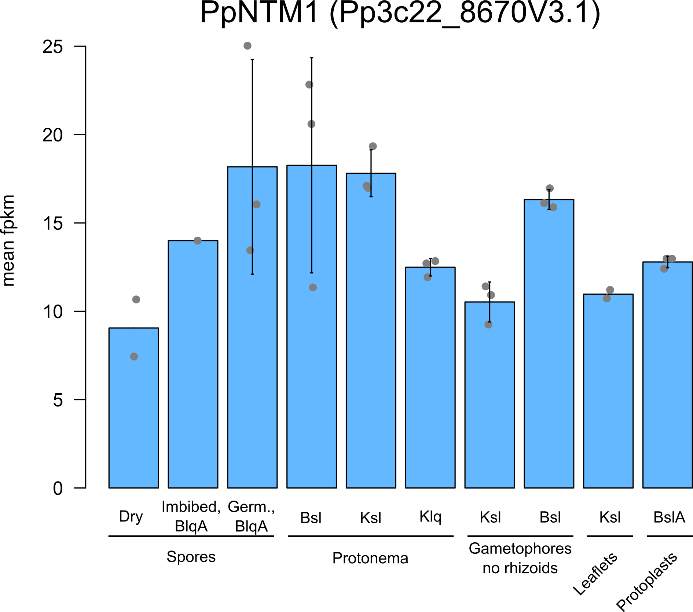


**Figure S6** **Expression levels of PpNTM1 in different tissues and cell culture types of Physcomitrella.** Expression levels are represented as mean fpkm (fragments per kilobase million) values with standard deviation. Single datapoints represent biological replicates. All replicate data were downloaded from *PEATmoss* (https://peatmoss.plantcode.cup.uni-freiburg.de) and sampling and methods are described in Perroud et al. (2018) and Fernandez-Pozo et al. (2020). Abbreviations: B = BCD medium (Ashton et al. 1979); K = Knop medium (Reski and Abel 1985); A =ammonium tartrate; lq = liquid; sl = solid; Germ. = germinating.


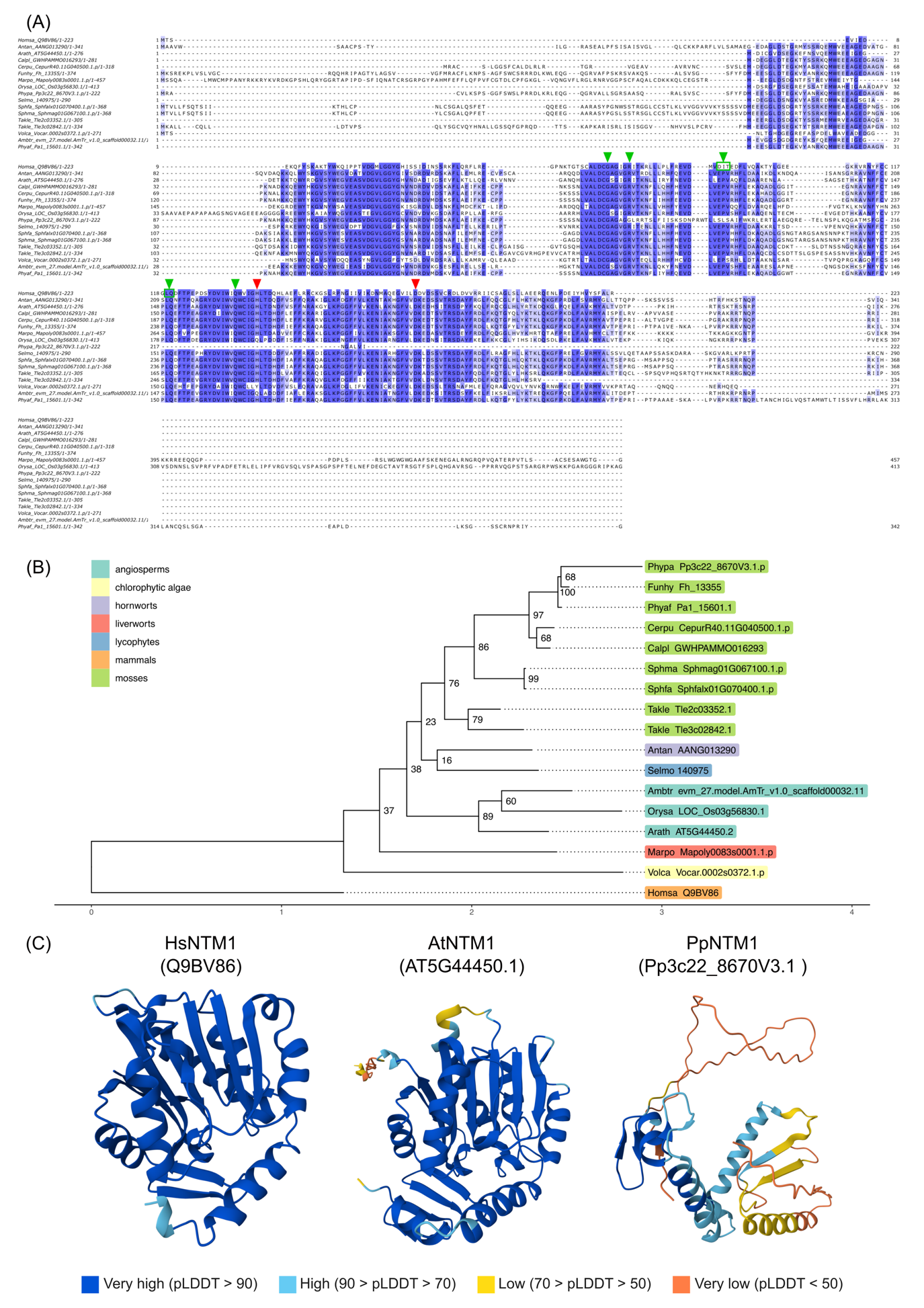


**Figure S7 Multiple Sequence alignment of selected NTM1 sequences, phylogenetic reconstruction and AlphaFold structures.** (**A**) Aligned homologous NTM1 amino acid sequences of human (Homsa), *Anthoceros angustus* (Antan), *Amborella trichopoda* (Ambtr), *Arabidopsis thaliana* (Arath), *Calohypnum plumiforme* (Calpl), *Ceratodon purpureus* (Cerpu), *Funaria hygrometrica* (Funhy), *Marchantia polymorpha* (Marpo), *Oryza sativa* (Orysa), *Physcomitrellopsis africana* (Phyaf), *Selaginella moellendorffii* (Selmo), *Sphagnum fallax* (Sphfa), *Sphagnum magellanicum* (Sphma), *Takakia lepidozioides* (Takle), *Volvox carteri* (Volca), and Physcomitrella (Phypa). Residues involved in binding of S-adenosyl-methionine (SAM) are indicated of HsNTM1 with red arrows (Dong et al. 2015; Wu et al. 2015), residues involved in target deprotonation are marked with green arrows (Dong et al. 2015; Wu et al. 2015). (**B**) Maximum likelihood tree of aligned NTM1 proteins with bootstrap support values at internal nodes and color-coded groups of taxa. (**C**) Structures of human, Arabidopsis, and Physcomitrella NTM1 predicted with AlphaFold (Jumper et al. 2021; Varadi et al. 2024). Structures were accessed via UNIPROT. The prediction accuracy of the models is indicated by color representing the per-residue model confidence score (pLDDT: predicted local distance difference test; Varadi et al. 2024).
